# Out-of-Hospital Cardiac Arrest as the First Manifestation of ALCAPA in a 9-Week-Old Infant: Survival to Discharge After Emergency Repair and VA-ECMO

**DOI:** 10.3390/jcm15218476

**Published:** 2026-10-30

**Authors:** Grzegorz Wróbel, Jakub Gruszka, Piotr Lewitowicz, Olga Adamczyk-Gruszka

**Affiliations:** 1 Department of Clinical and Experimental Pathology, Institute of Medical Sciences, Jan Kochanowski University, 25-516 Kielce, Poland; 2 2nd Department of Obstetrics and Gynecology, Faculty of Medicine, Medical University of Warsaw, 02-091 Warsaw, Poland; 3 Department of Gynecology and Obstetrics, Institute of Medical Sciences, Jan Kochanowski University, 25-516 Kielce, Poland

**Keywords:** anomalous left coronary artery from the pulmonary artery (ALCAPA), out-of-hospital cardiac arrest, infant, echocardiography, venoarterial extracorporeal membrane oxygenation (VA-ECMO), coronary reimplantation, multiorgan failure

## Abstract

**Background and Clinical Significance:** Anomalous origin of the left coronary artery from the pulmonary artery (ALCAPA) usually presents with heart failure at 2–3 months of age; cardiac arrest as the first manifestation is rare and often recognized only at autopsy. **Case Presentation:** A previously healthy girl collapsed at home at 65 days of age. After dispatcher-assisted parental resuscitation, the emergency team found asystole and achieved return of spontaneous circulation after approximately 4 min of professional resuscitation; the total low-flow time was not recorded. She was in cardiogenic shock (left ventricular ejection fraction 19.8%, high-sensitivity troponin T 2620 ng/L) with acute kidney injury and coagulopathy consistent with disseminated intravascular coagulation. Echocardiography showed a dilated right coronary artery and no flow from the aorta into the left coronary artery. Emergency repair on day 1 was followed by venoarterial extracorporeal membrane oxygenation (VA-ECMO) for 4 days and continuous renal replacement therapy. The Simpson ejection fraction reached about 50% by day 9, fell to 37–40% during respiratory deterioration and was 45–50% on day 27; mitral regurgitation, graded as massive during VA-ECMO, regressed to trace without valve surgery. She was discharged on day 42 with impaired diastolic indices; no formal neurological assessment was documented. **Conclusions:** ALCAPA should be considered in every infant with unexplained cardiac arrest, and post-resuscitation echocardiography should assess the coronary origins. Emergency repair with VA-ECMO can achieve survival to discharge despite multiorgan failure, but long-term cardiac and neurodevelopmental follow-up is required.

## 1. Introduction

Anomalous origin of the left coronary artery from the pulmonary artery (ALCAPA) is a rare congenital coronary anomaly, first described clinically by Bland, White and Garland in 1933 [1]. It occurs in approximately 1 in 300,000 live births and accounts for 0.24–0.46% of congenital heart disease; without surgical correction, up to 90% of affected infants die within the first year of life [2,3].

After birth, the physiological fall in pulmonary vascular resistance (PVR) reverses flow in the anomalous left coronary artery (LCA) into the low-pressure pulmonary trunk (coronary steal). The left ventricle (LV) then depends on collateral flow from the right coronary artery (RCA), and ischemic symptoms typically appear at 2–3 months of age [2]. Survival after surgical repair now exceeds 90% [4,5], and LV systolic function recovers in most survivors [6,7], but the diagnosis is often delayed: the anomalous origin is clearly visualized in only about half of echocardiographic examinations [6], and 49–74% of children are misdiagnosed before referral or at the initial echocardiogram, most often as dilated cardiomyopathy [8,9].

Cardiac arrest is an uncommon first manifestation of ALCAPA in infancy: in a multicenter cohort of 248 patients, it was the presenting event in only 1% of infants, compared with 11% of patients repaired at 1 year of age or later [10], and unrecognized cases may present as sudden unexpected infant death and be diagnosed only at autopsy [11,12]. Among children, out-of-hospital cardiac arrest (OHCA) is most frequent and has the poorest outcome in infancy, with 30-day survival of only 17% in a recent national cohort [13,14], and because cardiac arrest in infants is usually not of primary cardiac origin [15], a treatable coronary anomaly can easily be overlooked during resuscitation and post-arrest care.

We report a 9-week-old girl with an uneventful perinatal history in whom OHCA was the first manifestation of ALCAPA and who survived to hospital discharge after emergency coronary repair, venoarterial extracorporeal membrane oxygenation (VA-ECMO) and continuous renal replacement therapy (CRRT). The report follows the CARE guidelines [16].

## 2. Case Presentation

### 2.1. Perinatal History and Presentation

The girl was born by spontaneous vaginal delivery after an uncomplicated pregnancy, with a birth weight of 3400 g and an Apgar score of 10. Prenatal ultrasound examinations had not revealed any cardiac abnormality, and the family history was negative for heart disease.

At 65 days of age (day 0), she suddenly became unresponsive, hypotonic and cyanotic at home and stopped breathing. The parents started cardiopulmonary resuscitation (CPR) following telephone instructions from the dispatcher. On arrival, the emergency medical service (EMS) team found asystole, continued CPR and achieved return of spontaneous circulation (ROSC) after approximately 4 min of professional resuscitation. The interval from collapse to the start of parental CPR (no-flow time) and the duration of dispatcher-assisted CPR before EMS arrival were not recorded, and EMS rhythm strips and dispatch records were not available to the authors; the 4 min interval therefore represents only the professional component of the low-flow time, and the total duration of the arrest is unknown.

On admission to the neonatal intensive care unit of the regional referral hospital, the infant was ventilated with a bag-mask device. Her heart rate was 160/min and blood pressure 69/42 mmHg, with signs of poor peripheral perfusion (pallor, cold extremities and mottled skin of the chest). The abdomen was distended, and the liver was palpable 4 cm below the right costal margin. Spontaneous breathing (50/min) was ineffective, with high airway resistance. After intubation under midazolam and ketamine, diffuse wheezes and basal crackles were heard; oxygen saturation rose from 79–80% to 98% within minutes of ventilation and reached 100% at a fraction of inspired oxygen (FiO_2_) of 0.7.

Bedside echocardiography performed by a pediatric cardiologist raised the suspicion of ALCAPA, and a dobutamine infusion was started. The infant was transferred on the same day by a neonatal transport team to a tertiary pediatric cardiac surgery center.

Laboratory tests at the referral hospital (Table 1) showed acute myocardial injury, with a high-sensitivity troponin T (hs-TnT) concentration of 2620 ng/L and N-terminal pro-B-type natriuretic peptide (NT-proBNP) above the upper measuring limit of the assay (35,000 pg/mL). Post-arrest multiorgan injury was evident: ischemic liver injury with hyperammonemia, acute kidney injury (AKI) with hyperkalemia and hyperphosphatemia, severe anemia (hemoglobin 6.8 g/dL) and a coagulopathy with an International Society on Thrombosis and Haemostasis (ISTH) score of at least 5 points, consistent with overt disseminated intravascular coagulation (DIC), although prothrombin time could not be determined [17,18]. C-reactive protein was normal, whereas procalcitonin was increased (2.81 ng/mL). The creatine kinase MB isoenzyme (CK-MB), reported as catalytic activity (U/L), accounted for 65% of total creatine kinase (CK) activity.

### 2.2. Diagnosis at the Cardiac Surgery Center

On admission to the cardiac surgery center, the infant was in critical condition, intubated and ventilated with an FiO_2_ of 0.9, receiving high-dose inotropes, with features of pulmonary edema and diuretic-dependent oliguria; arterial pH was 7.25 and lactate 4.0 mmol/L. Body weight was 4.5 kg and length 60 cm.

Transthoracic echocardiography (Table 2) showed situs solitus with normal systemic and pulmonary venous connections, concordant atrioventricular and ventriculoarterial connections, a left aortic arch, intact atrial and ventricular septa and no patent ductus arteriosus. The LV was markedly dilated (end-diastolic dimension 36.7 mm, end-systolic dimension 33.3 mm, fractional shortening 9.3%), with thin walls (interventricular septum 3.0 mm, posterior wall 3.5 mm), flattened septal motion and severely reduced systolic function: the ejection fraction (EF) was 20% by the Teichholz method and 19.8% by the Simpson method. Mitral regurgitation (MR) was mild, with two jets.

The RCA arose from the right coronary sinus and was dilated to 2.5 mm, with antegrade flow, whereas no flow from the aorta into the LCA could be demonstrated. Neither the origin of the LCA from the pulmonary trunk nor retrograde flow into the pulmonary trunk was imaged, and flow in the pulmonary trunk was reported as normal; ALCAPA was therefore diagnosed presumptively, from these indirect signs and the clinical picture, and the infant was referred for emergency surgery without computed tomography (CT) angiography or catheterization. The electrocardiogram showed sinus tachycardia with ST-segment depression in leads I, aVL and V5–V6 and deep Q waves in V5–V6, consistent with anterolateral ischemia. The chest radiograph showed cardiomegaly and atelectatic consolidations in the left upper and right middle lung fields.

### 2.3. Surgery and Postoperative Course

**Figure 1:**
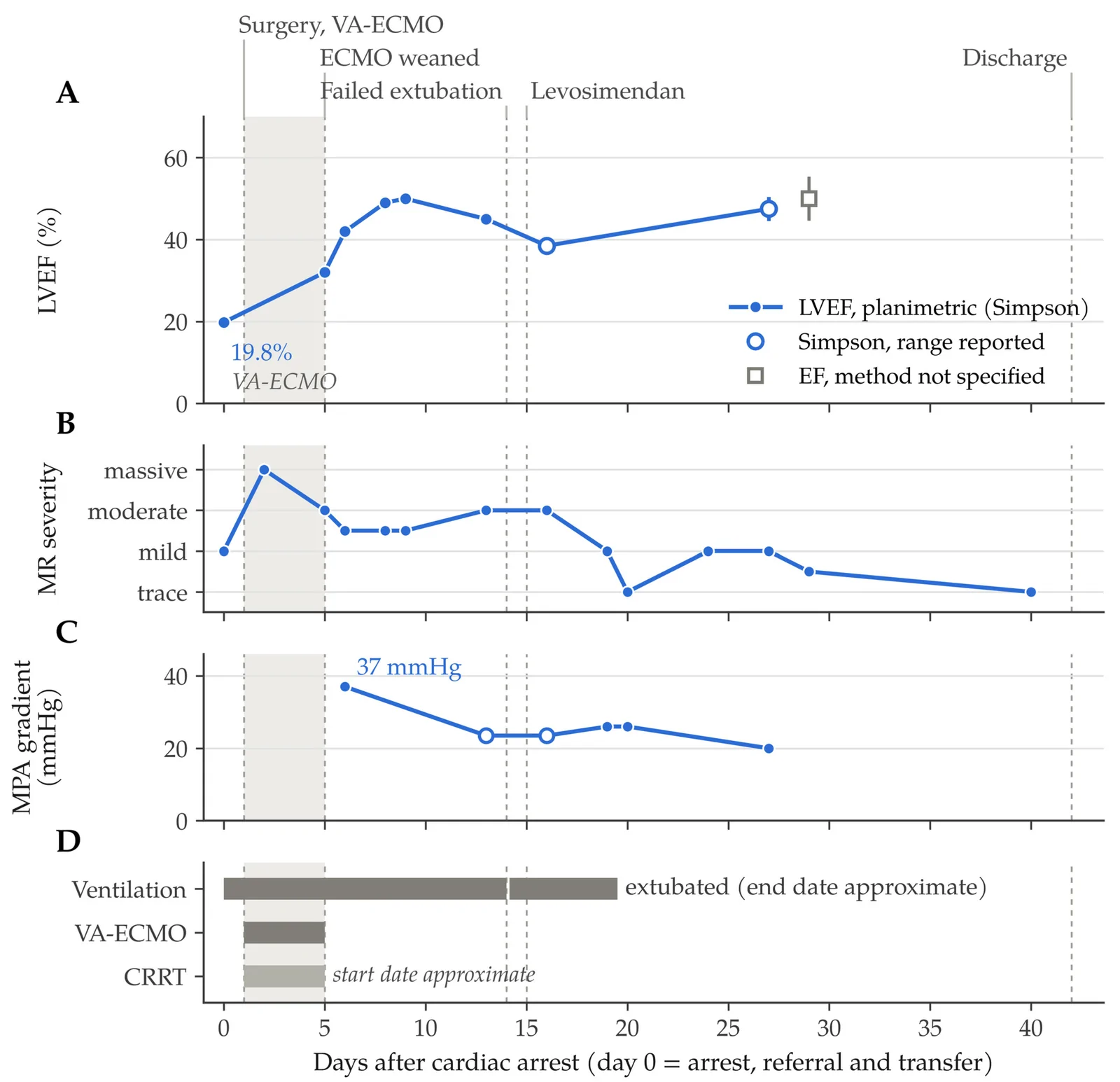
Clinical course after cardiac arrest (day 0 = day of arrest, referral and transfer). (**A**) Left ventricular ejection fraction (LVEF) assessed consistently by the planimetric Simpson method (circles; open circles with bars indicate ranges given in the reports); the open square on day 29 marks an EF of 45–55% reported without specification of the method. M-mode (Teichholz) estimates are not plotted because they are not interchangeable with Simpson values in the presence of regional wall-motion abnormalities and paradoxical septal motion (see Table 2). (**B**) Severity of mitral regurgitation (MR), graded qualitatively on color Doppler as stated in the reports; intermediate points represent grades reported as mild-to-moderate or trace-to-mild. (**C**) Peak systolic gradient across the main pulmonary artery (MPA); open circles with bars indicate reported ranges. (**D**) Organ support: mechanical ventilation (the gap marks the failed extubation on day 14; the end of ventilation is dated from serial chest radiographs), venoarterial extracorporeal membrane oxygenation (VA-ECMO) and continuous renal replacement therapy (CRRT; start date approximate). The shaded band marks VA-ECMO support, and dashed vertical lines indicate key clinical events.

Despite elevated inflammatory markers, thrombocytopenia and AKI with serum creatinine above 100 µmol/L (77 µmol/L at the referral hospital), life-saving surgery was performed on day 1. The final diagnosis recorded in the discharge summary of the cardiac surgery center after the operation was anomalous origin of the LCA from the pulmonary artery, and the procedure was described as an uncomplicated correction with complete revascularization under cardiopulmonary bypass; the original operative report was not available. Postoperative echocardiography showed the LCA arising from the aortic coronary sinus, consistent with direct reimplantation (coronary button transfer). Because of hemodynamic instability due to severe LV dysfunction after the repair, VA-ECMO was instituted at the end of the operation, and the sternum was left open. ECMO cannulae were visible on chest radiographs, but the cannulation configuration, pump flow, anticoagulation protocol and any left heart decompression were not described in the available documentation; the atrial septum was intact on all postoperative echocardiograms, so no atrial septostomy was performed. The clinical course is summarized in Figure 1.

During VA-ECMO, hypotension persisted despite catecholamines and required vasopressin. Broad-spectrum antibiotics (piperacillin-tazobactam and teicoplanin) were administered. Echocardiography on day 2 showed a dilated LV with an akinetic posterior wall, residual septal motion limited to the basal and mid segments, hyperechogenic posterior papillary muscles and MR graded as massive; the RV was compressed, with a mid-cavity gradient of 11 mmHg. The reimplanted LCA was visible from its aortic origin along its epicardial course to the apex, with preserved flow on color Doppler. An 11 × 9 mm hyperechogenic area adjacent to the LCA origin, reported on days 2 and 5, was not characterized further and was no longer described from day 6 onwards, while flow in the LCA remained visible on every subsequent examination (Table 2); because the area was much larger than the coronary lumen and LCA flow was preserved, an extraluminal structure, such as a perivascular hematoma or hemostatic material at the anastomosis, appears more likely than an intraluminal thrombus.

VA-ECMO was discontinued on day 5, after 4 days of support, once hemodynamics had stabilized, LV systolic function had improved (Simpson EF approximately 32%, MR reduced to moderate) and spontaneous diuresis had returned; these were the criteria for decannulation recorded in the discharge summary. Dopamine and milrinone were continued, whereas epinephrine and vasopressin were gradually withdrawn.

Oligoanuria had developed during VA-ECMO despite vasoactive support, and CRRT (hemofiltration), started on day 1, was discontinued together with VA-ECMO on day 5, after spontaneous diuresis had returned; no further renal replacement therapy was required. Urine output before CRRT was 0.2 mL/kg/h. Arterial pH had normalized by day 2, lactate peaked at 8.5 mmol/L on day 1 and normalized by day 3, and serum creatinine peaked at 130 µmol/L on day 1 and was 30 µmol/L at discharge. Both kidneys remained hyperechogenic on ultrasound, with preserved perfusion on color Doppler. Aspartate aminotransferase activity returned to the reference range by day 6, and transient ascites, present on days 13–15, had resolved by day 19, although hepatomegaly persisted on that examination.

By day 9, the Simpson EF had reached approximately 50%. The subsequent course was complicated by pulmonary edema, atelectasis of the left lung and later of the right upper lobe requiring intensive chest physiotherapy, and abdominal distension with feeding intolerance that required total parenteral nutrition. Between days 13 and 16, the Simpson EF declined to 37–40% and MR increased to moderate. Extubation on day 14 failed within a few hours because of respiratory failure, and levosimendan was infused on day 15. Bronchoscopy on day 16 revealed abundant frothy, blood-stained secretions, predominantly on the left, without airway obstruction. LV function then improved again, MR decreased to trace by day 20, and the infant was extubated (the endotracheal tube was last documented on day 19), followed by a gradual transition to enteral feeding. The last Simpson EF before discharge was 45–50% on day 27, and an EF of 45–55% was reported on day 29 without specification of the method; no EF was reported on the final echocardiogram on day 40.

The peak gradient of turbulent flow in the main pulmonary artery (MPA) decreased from 37 mmHg on day 6 to 20 mmHg on day 27. Serial echocardiograms showed hyperechogenic LV endocardium and papillary muscles. The LV posterior wall was reported as up to 12 mm on days 21 and 29 (8–9 mm in a second measurement on day 29), but as 5 mm on M-mode and 9–11 mm in the apical four-chamber view on day 27. The ratio of early mitral inflow velocity to early diastolic mitral annular velocity (E/e′) ranged from 12.5 to 24.9 on days 6–21, during mechanical ventilation and inotropic support, and from 7 to 17 on days 27–40, with fusion or reversal of the E and A waves (Table 2). The treating team interpreted these findings as impaired LV diastolic function and considered it the most likely cause of the poor weight gain (4.4 kg at discharge). Color Doppler showed good flow in the left main and left anterior descending (LAD) arteries before discharge.

The infant was discharged home in good general condition on day 42, on age-appropriate enteral feeding and receiving carvedilol 1.5 mg, captopril 2 mg and spironolactone 2 mg, each three times daily, and aspirin 10 mg once daily, with multidisciplinary follow-up arranged at the pediatric cardiology, nephrology, neurology and gastroenterology outpatient clinics. Neurological status at discharge was documented only as good general condition: no formal neurological examination, assessment for seizures, electroencephalography or neuroimaging was recorded during the hospital stay, no antiseizure medication was prescribed, and the infant was referred to the pediatric neurology clinic for diagnostic evaluation. Data on growth, neurodevelopment and cardiac status after discharge were not available to the authors; the outcome is therefore reported as survival to hospital discharge.

## 3. Discussion

This case illustrates three clinically relevant aspects of ALCAPA: cardiac arrest as the first manifestation at the typical age of decompensation, a post-arrest laboratory profile that may obscure the cardiac diagnosis, and survival to hospital discharge after emergency repair with VA-ECMO despite multiorgan failure.

### 3.1. Literature Review

The literature was identified by a targeted narrative search of PubMed/MEDLINE and Scopus (reports published between January 2016 and September 2026, searched in October 2026) using combinations of the terms “anomalous left coronary artery from the pulmonary artery”, “ALCAPA”, “Bland-White-Garland syndrome”, “cardiac arrest”, “ventricular fibrillation”, “sudden death”, “extracorporeal membrane oxygenation”, “mitral regurgitation” and “echocardiography”, supplemented by screening of the reference lists of the retrieved articles.

### 3.2. Cardiac Arrest in Infancy: Diagnostic and Forensic Implications

Cardiac arrest as the first manifestation is more frequent in late presenters, whose better-developed collaterals permit survival beyond infancy at the cost of chronic ischemia and arrhythmias [6,10]. Infants usually present with heart failure [2] or, rarely, acute myocardial infarction [23], and the few infant reports of an arrhythmic presentation document diagnostic failure: ALCAPA was missed on several echocardiograms in a neonate with ventricular fibrillation [24] and was diagnosed only by postmortem CT and autopsy in a 5-month-old girl who died suddenly [11].

Among the reports identified, none documented in comparable detail survival to discharge after OHCA as the first manifestation in early infancy, followed by emergency repair and postoperative VA-ECMO, together with serial echocardiographic data up to discharge (Table 2, Figure 1).

Survival after infant OHCA was 4.9% in the data summarized in the 2020 American Heart Association (AHA) guidelines, which list coronary thrombosis among the reversible causes to be sought and recommend considering multiple factors when predicting outcome after ROSC [14]; the 2025 AHA and American Academy of Pediatrics guidelines state that echocardiography may be considered to identify treatable causes of cardiac arrest in infants and children [15]. In our patient, bedside echocardiography after ROSC raised the suspicion of ALCAPA on the day of the arrest; we suggest that post-ROSC echocardiography in an infant with unexplained arrest should include deliberate visualization of both coronary origins and color Doppler interrogation of the pulmonary trunk for retrograde coronary flow.

Fatal cases show the forensic relevance of the anomaly. Anomalous origin of a coronary artery from the pulmonary artery is listed as a certain cause of sudden cardiac death in the guidelines of the Association for European Cardiovascular Pathology [25] and is among the most lethal coronary variants recognized at autopsy [12], and postmortem CT can show the myocardial calcification of chronic ischemia [11]. The coronary ostia should therefore be examined systematically in every infant who dies suddenly and unexpectedly.

### 3.3. The Perinatal “Silent Window” and Early Recognition

**Figure 2:**
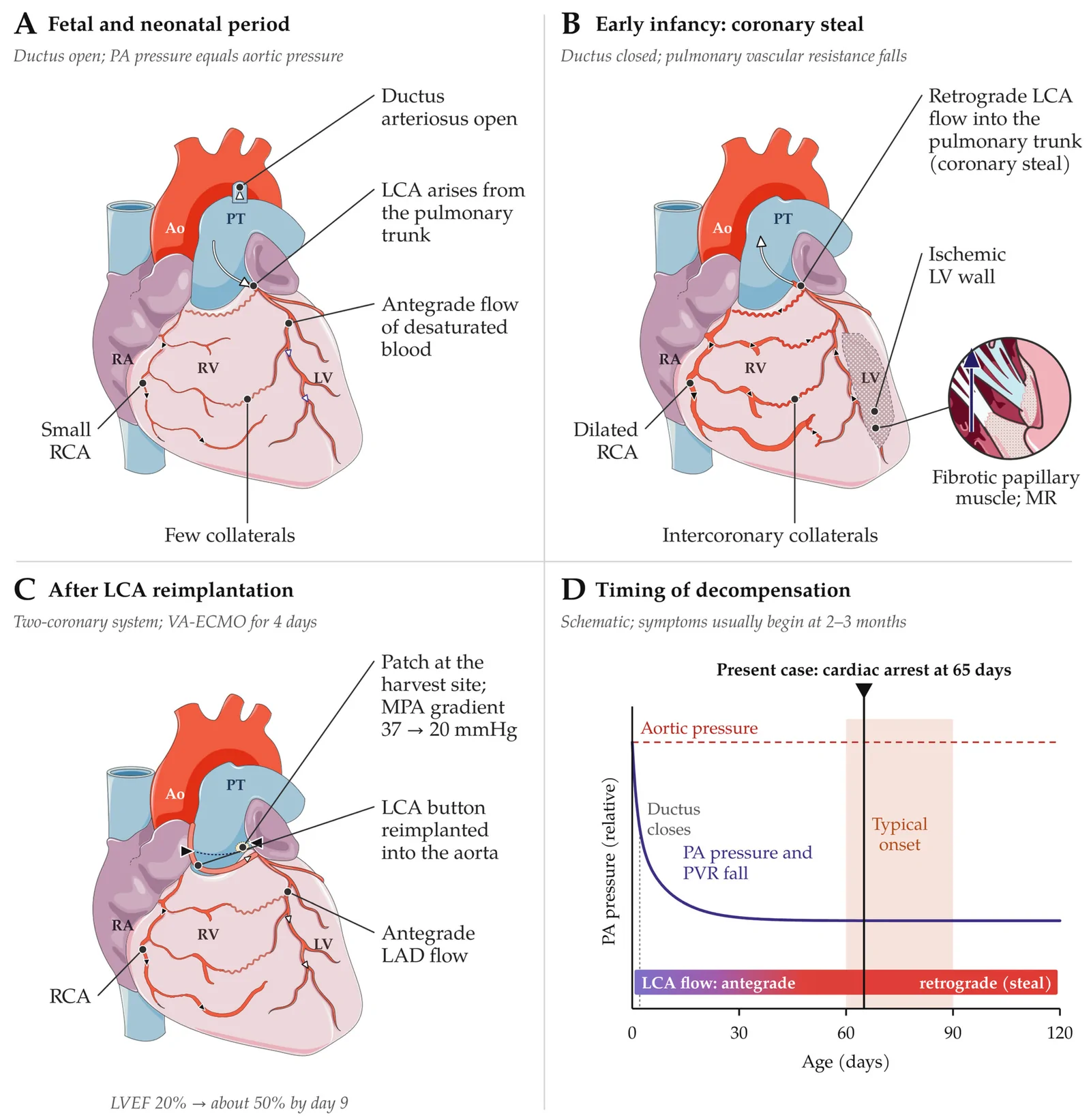
Pathophysiology and timing of decompensation in anomalous left coronary artery from the pulmonary artery (ALCAPA); schematic anterior view of the heart. (**A**) Fetal and neonatal period: equal pressures in the pulmonary trunk (PT) and aorta (Ao) allow antegrade perfusion of the anomalous left coronary artery (LCA) with desaturated blood. (**B**) Early infancy: after ductal closure and the fall in pulmonary vascular resistance (PVR), blood from the dilated right coronary artery (RCA) reaches the LCA branches through collaterals and drains retrogradely into the PT (coronary steal), causing ischemia of the left ventricular (LV) wall and papillary muscles (inset; MR, mitral regurgitation). (**C**) After surgical repair: direct reimplantation of the LCA into the aorta (dashed segment, course behind the PT) restores antegrade flow; reconstruction of the harvest site may narrow the main pulmonary artery (MPA). The technique shown is the one inferred from postoperative echocardiography, because the operative report was not available; in the present patient, the left ventricular ejection fraction (LVEF) reached about 50% by day 9 but subsequently fluctuated (Figure 1A). (**D**) Timing of decompensation: schematic pressure relationship between the aorta and the pulmonary artery (PA) over time; the present patient had cardiac arrest at 65 days, at the typical age of symptom onset. LAD, left anterior descending artery; RA, right atrium; RV, right ventricle; VA-ECMO, venoarterial extracorporeal membrane oxygenation. The figure was drawn by the authors in Inkscape 1.3.2; the heart drawings were adapted from Servier Medical Art https://smart.servier.com (accessed on 26 October 2026), licensed under CC BY 4.0 (https://creativecommons.org/licenses/by/4.0/).

The timing of the arrest, at 65 days of age, is typical (Figure 2D). In fetal life, the LCA is perfused antegradely with desaturated blood at systemic pressure (Figure 2A) [2], consistent with the uneventful neonatal course of our patient; as PVR falls, flow in the LCA reverses into the pulmonary trunk, and the LV becomes dependent on collaterals from the RCA (Figure 2B), which are insufficient in the infantile form of the disease, accounting for about 85% of cases [2,6]. Ischemia is precipitated by the oxygen demand of crying and feeding, and symptoms usually develop at 2–3 months of age [2]. In our patient, severe anemia may have further reduced myocardial oxygen delivery after the arrest.

ALCAPA escapes perinatal screening, because the coronary arteries are not assessed in the views recommended for routine fetal cardiac screening [26] and newborn pulse oximetry targets critical congenital heart disease with hypoxemia [27]. Detection therefore depends on subtle signs in the first weeks of life, such as irritability, pallor or sweating during feeding (infant angina), tachypnea, poor weight gain or a new murmur of MR [2]; in retrospect, our patient gained only about 1.1 kg in 9 weeks. Such signs should prompt electrocardiography and echocardiography with deliberate visualization of both coronary origins: anterolateral Q waves are present in about two-thirds of children with ALCAPA, but 8% have a normal electrocardiogram [28], and indirect echocardiographic markers, such as reversed LCA flow, collateral flow and RCA dilation, are more reliable than direct imaging of the anomalous origin [6].

Before surgery, our patient had a dilated RCA, no demonstrable flow from the aorta into the LCA, severe LV dysfunction and MR, whereas neither the anomalous origin nor reversed LCA flow into the pulmonary trunk was imaged; the diagnosis therefore remained presumptive until surgery. The features that distinguish ALCAPA from its most frequent mimics are summarized in Table 3. When echocardiography is inconclusive and the clinical condition allows, CT angiography is the imaging modality of choice for coronary anomalies, and cardiac magnetic resonance imaging (MRI) with late gadolinium enhancement (LGE) characterizes myocardial scar [3].

The hyperechogenic posterior papillary muscles and endocardium and the akinetic posterior wall in our patient are compatible with ischemic injury and possibly with secondary endocardial fibroelastosis (EFE) [30], resembling the acute-on-chronic ischemic substrate described at autopsy by Fukuda et al. [11]; however, echocardiography cannot distinguish edema, necrosis, fibrosis and fibroelastosis, and no tissue characterization by cardiac MRI or histology was available.

### 3.4. Interpretation of Laboratory Findings After Cardiac Arrest

The laboratory abnormalities reflected both the underlying anomaly and the post-cardiac arrest syndrome, which comprises brain injury, myocardial dysfunction, a systemic ischemia–reperfusion response and the persistent precipitating pathophysiology [31], in this case the uncorrected coronary steal. The hs-TnT concentration, approximately 33 times the age-specific 99th percentile [21], confirmed marked myocardial injury, but after cardiac arrest, reperfusion and shock a single value cannot be used to estimate the extent of necrosis. Similarly, NT-proBNP exceeded the upper measuring limit of the assay and thus more than 54 times the 95th percentile for infants, whose reference values are several times higher than adult limits [22].

In contrast, CK-MB activity could not be interpreted. Its reporting in U/L suggests an immunoinhibition assay, which assumes the absence of the creatine kinase BB isoenzyme (CK-BB), so that CK-BB released from the hypoxic brain or bowel, or macro-CK, falsely increases the result and can even make CK-MB exceed total CK [19]. In myocardial infarction, CK-MB usually accounts for 6–25% of total CK activity and generally does not exceed 30% [20]; a ratio of 65% therefore suggests analytical interference, although the method was not stated in the laboratory report and no isoenzyme electrophoresis or mass assay was performed to confirm it.

Leukocytosis and increased procalcitonin with normal C-reactive protein are compatible with the inflammatory response after resuscitation. Early coagulopathy occurs in 46% of children after OHCA [32], and the coagulofibrinolytic changes after cardiac arrest meet the ISTH definition of DIC [33]. In our patient, platelet count, D-dimer and fibrinogen alone reached the ISTH threshold of 5 points [17], so the missing prothrombin time could only have led to an underestimation; because the score was developed in adults and has been applied in children mainly in single-center series [18], overt DIC should nevertheless be regarded as a supportive rather than definitive diagnosis. None of these abnormalities is a reason to postpone surgery, because the coronary steal persists until a two-coronary system is restored.

### 3.5. Emergency Repair and Mechanical Circulatory Support

Direct reimplantation is the preferred technique in infants [2,4]. The use of mechanical circulatory support (MCS) after repair varies widely, from none in the 40-year experience of Lange et al. [34] to 7–8% in recent Chinese and Brazilian cohorts [35,36] and 36% in the Melbourne series [37].

In the Extracorporeal Life Support Organization (ELSO) registry, 74% of 163 children with ALCAPA supported with extracorporeal membrane oxygenation (ECMO) survived to discharge, and lower pre-ECMO pH and a longer time to ECMO were associated with mortality [38]. This compares favorably with 40–60% survival after pediatric post-cardiotomy ECMO in general [39], possibly because much of the ischemic myocardium in ALCAPA is hibernating or stunned rather than irreversibly damaged.

The ELSO guidelines list ALCAPA among the indications for ECMO in pediatric cardiac failure, note that myocardial improvement after cardiotomy may occur within 24 h to 5 days, and address left heart decompression [40]. Because VA-ECMO increases LV afterload, a poorly contracting ventricle with severe MR may distend, with pulmonary edema; the rationale for LV unloading has been reviewed mainly in adults [41]. In our patient, the LV was dilated and MR was graded as massive on day 2, yet support could be discontinued after 4 days, once LV function and diuresis had improved; the Simpson EF reached approximately 50% by day 9, but this early improvement was not sustained.

The secondary decline in LV function, with pulmonary edema, blood-stained airway secretions and a failed extubation, shows how vulnerable the recovering ventricle is to changes in loading conditions during weaning from mechanical ventilation; younger age, AKI, higher lactate concentrations and tachycardia predict prolonged ventilation after ALCAPA repair [42]. A Cochrane network meta-analysis found high-certainty evidence that levosimendan reduces low cardiac output syndrome after surgery for congenital heart disease [43], but the improvement observed after its infusion in our patient cannot be attributed to the drug alone.

### 3.6. Mitral Regurgitation, Pulmonary Artery Gradient and Myocardial Recovery

Mitral regurgitation is the most frequent indication for reoperation after ALCAPA repair [4]. In our patient, MR was graded qualitatively on color Doppler, without quantitative indices such as vena contracta width or proximal isovelocity surface area, and its mechanism was not described in the reports. It was mild before surgery, was graded as massive on day 2 during VA-ECMO, when the LV was dilated, the posterior wall akinetic and the posterior papillary muscles hyperechogenic, and regressed to trace within three weeks without valve surgery. This suggests ischemic functional MR, but because MR severity depends strongly on ventricular geometry and loading conditions, which changed profoundly before, during and after VA-ECMO, the recorded grades do not reflect changes in the valve lesion alone.

In a meta-analysis of 53 studies (3851 patients), concomitant mitral surgery did not affect mortality, MR improved after revascularization in most patients, and about 10% required a second mitral operation [5]; however, in a series of patients with moderate or severe MR, improvement was less frequent without concomitant repair (51.4% vs. 79.4%), and all late mitral operations occurred in that group [44]. A single favorable course therefore does not show that severe ischemic MR can routinely be left untreated; the decision depends on its mechanism and severity and on the expected recovery of the papillary muscles, and mitral function requires long-term follow-up, as late mitral surgery may be needed decades after repair [37].

Two further findings determine surveillance. First, the MPA gradient, which decreased from 37 to 20 mmHg, most likely reflects turbulence at the reconstructed harvest site (Figure 2C); supravalvular pulmonary stenosis developed in 26.1% of children after reimplantation in one series [45] and was a leading cause of reoperation in another [46], although none occurred in a third [9], and CT is useful when stenosis is suspected [3].

Second, LV systolic function recovered only partially before discharge. By the Simpson method, the EF increased from 19.8% to approximately 50% by day 9, fell to 37–40% on day 16 and was 45–50% on day 27, and no EF was reported at discharge. M-mode (Teichholz) estimates, up to 77%, are shown only for completeness (Table 2), because the method extrapolates volume from a single basal dimension, which is invalid with an akinetic posterior wall, flat or paradoxical septal motion and a small, thick-walled cavity. In published series, the median time to recovery of LV systolic function ranged from 10 days to 5.3 months [9,45], and in a long-term study, the EF normalized in 92% of patients, but diastolic dysfunction persisted in 57% and was associated with heart failure or ventricular tachyarrhythmia [7].

Diastolic function was even more difficult to assess. E/e′ ratios of 20–25 were recorded during mechanical ventilation, inotropic support and changing preload, and lower values (7–17) on days 27–40. In infants, E/e′ is age-dependent and influenced by heart rate, fusion of the E and A waves and loading conditions, and a recent consensus statement of the European Association of Cardiovascular Imaging and the Association for European Paediatric and Congenital Cardiology cautions against applying adult-derived diastolic parameters across pediatric ages and loading conditions [47]. Corroborating data were limited to a left atrial-to-aortic root ratio of 1.58 on day 13, decreasing to 1.22 by day 19, and intermittent E/A reversal, so the findings are compatible with impaired LV relaxation but do not prove persistently elevated filling pressures. Likewise, the posterior wall thickness of up to 12 mm varied with the imaging view and may reflect edema or the inclusion of the hyperechogenic papillary muscles and endocardium rather than hypertrophy. Long-term surveillance should therefore include diastolic indices, myocardial deformation imaging and, when indicated, cardiac MRI, in addition to the EF [3,7].

### 3.7. Limitations

This report has limitations. It is a retrospective description of a single patient based on written hospital documentation; the original electrocardiogram, operative report, EMS records and echocardiographic recordings were not available, so the resuscitation timeline could be reconstructed only in part, the anomalous origin and the surgical technique could not be documented with images, and MR and diastolic function could not be remeasured. Details of VA-ECMO management (cannulation, flows, anticoagulation and LV decompression) were not recorded. Neurological status was not formally assessed before discharge, and no follow-up data were available, so the outcome is limited to survival to hospital discharge; longer resuscitation predicts mortality even after in-hospital cardiac arrest in adults [48], and although such data cannot be extrapolated to infant OHCA, the unknown total low-flow time precludes any inference on the neurological prognosis. Finally, the literature review was narrative, so infant cases published in journals not indexed in the searched databases may have been missed.

## 4. Conclusions

ALCAPA should be considered in every infant with unexplained collapse, cardiac arrest or severe LV dysfunction in the first months of life, because the falling PVR unmasks the anomaly at about 2–3 months of age, typically after a normal perinatal course. Cardiac arrest is a rare first manifestation, but post-ROSC echocardiography with targeted assessment of the coronary origins can establish the diagnosis on the day of the arrest; a dilated RCA and the absence of flow from the aorta into the LCA are the key clues, although the diagnosis may remain presumptive until surgery. After cardiac arrest, cardiac troponin interpreted against age-specific limits, rather than CK-MB activity, should be used to assess myocardial injury. Emergency repair, followed when necessary by VA-ECMO and CRRT, can achieve survival to discharge despite multiorgan failure, but recovery of LV function may be incomplete, and survivors require long-term surveillance of systolic and diastolic function, MR, coronary patency, pulmonary artery stenosis and neurodevelopment. At the autopsy of infants who die suddenly, the coronary ostia should be examined systematically.

## Figures and Tables

**Table 1:** Laboratory findings at presentation (regional referral hospital, day 0).

Parameter	Result	Reference Range	×URL	Interpretation
Myocardial injury and heart failure				
hs-TnT	2620 ng/L	0–14.1 (adult); ≈80 at 2 months ^a^	≈33 ^a^	Marked myocardial injury; extent of necrosis not estimable after arrest and shock
NT-proBNP	>35,000 pg/mL	0–125 (adult); 646 at 1 month–1 year ^b^	>54 ^b^	Above the upper measuring limit of the assay
CK	1588 U/L	20–180	8.8	Myocardial and other tissue injury
CK-MB activity	1028 U/L	0–25	41	CK-MB/CK ratio 65%; CK-BB or macro-CK interference possible, not confirmed [19,20]
Lactate dehydrogenase	3551 U/L	120–300	11.8	Multiorgan ischemic injury
Liver				
AST	792 U/L	15–60	13.2	Ischemic liver injury; possible myocardial contribution
ALT	276 U/L	13–45	6.1	Ischemic liver injury
Ammonia	263 µg/dL (154 µmol/L)	18.7–86.9 µg/dL	3.0	Hepatic dysfunction
Kidney and electrolytes				
Creatinine	0.87 mg/dL (77 µmol/L)	0.16–0.39 mg/dL	2.2	Acute kidney injury
Potassium	6.44 mmol/L	3.50–5.20	–	Hyperkalemia
Phosphate	8.36 mg/dL (2.70 mmol/L)	4.00–7.00 mg/dL	–	Hyperphosphatemia
Magnesium	3.12 mg/dL (1.28 mmol/L)	1.50–2.20 mg/dL	–	Hypermagnesemia
Total calcium	8.10 mg/dL (2.02 mmol/L)	9.00–11.00 mg/dL	–	Hypocalcemia
Hematology and coagulation				
White blood cells	20.83 × 10^9^/L	5.50–18.00	–	Leukocytosis with neutrophilia (13.64 × 10^9^/L)
Hemoglobin	6.8 g/dL	10.0–18.0	–	Severe anemia (hematocrit 21.5%; MCV 98.2 fL)
Platelets	71 × 10^9^/L	150–600	–	Thrombocytopenia (ISTH: 1 point)
APTT	91.8 s, then unmeasurable	25.4–36.9 s	–	Severe coagulopathy
Thrombin time	Unmeasurable	11.0–17.8 s	–	Severe coagulopathy
Fibrinogen	0.36 g/L	2.00–3.93 g/L	–	<1 g/L (ISTH: 1 point)
D-dimer	43,142 µg/L	0–500	86	Marked increase (ISTH: 3 points); ISTH score ≥5 without prothrombin time: consistent with overt DIC
Inflammation				
C-reactive protein	<0.6 mg/L	0.6–5.0	–	Normal
Procalcitonin	2.81 ng/mL	<0.50	–	Increased; nonspecific after cardiac arrest

Values obtained at the regional referral hospital on day 0 (day of cardiac arrest); reference ranges as provided by the laboratory, which for some analytes are not age-specific; SI units in parentheses. ^a^ The laboratory limit for hs-TnT (14.1 ng/L) is derived from adults; the multiple refers to the age-specific 99th percentile of approximately 80 ng/L at 60 days of age, determined with the same assay platform [21]. ^b^ The laboratory range for NT-proBNP (0–125 pg/mL) applies to adults; the multiple refers to the 95th percentile of 646 pg/mL in infants aged 1 month to 1 year [22]. Sodium (139 mmol/L), urea (26 mg/dL) and gamma-glutamyl transferase (59 U/L) were within reference ranges. Prothrombin time could not be obtained and was not scored; because platelet count, D-dimer and fibrinogen yielded 5 points, the ISTH threshold for overt DIC was reached irrespective of prothrombin time. CK-MB was reported as catalytic activity (U/L); the assay method was not stated in the laboratory report, and CK isoenzyme electrophoresis was not performed. A dash indicates that the multiple of the URL was not calculated. ALT, alanine aminotransferase; APTT, activated partial thromboplastin time; AST, aspartate aminotransferase; CK, creatine kinase; CK-BB, creatine kinase BB isoenzyme; CK-MB, creatine kinase MB isoenzyme; DIC, disseminated intravascular coagulation; hs-TnT, high-sensitivity troponin T; ISTH, International Society on Thrombosis and Haemostasis; MCV, mean corpuscular volume; NT-proBNP, N-terminal pro-B-type natriuretic peptide; URL, upper reference limit.

**Table 2:** Serial transthoracic echocardiography.

Day (Setting)	EF Simpson (%)	EF/FS M-Mode (%) ^d^	LVEDd/LVESd (mm)	IVSd/LVPWd (mm)	MR	MPA PG (mmHg)	Other Findings
0, before surgery	19.8	20/9.3 ^a^	36.7/33.3	3.0/3.5	Mild (2 jets)	Normal flow	RCA 2.5 mm from right sinus; no flow from the aorta into the LCA; LCA origin not imaged; flattened septal motion
2, VA-ECMO	Reduced	n.r.	n.r.	n.r.	Massive	n.r.	Dilated LV; akinetic posterior wall; hyperechogenic posterior papillary muscles; LCA from aortic sinus with flow; 11 × 9 mm hyperechogenic area near LCA origin; RV mid-cavity gradient 11 mmHg
5, ECMO weaning	≈32	n.r.	n.r.	n.r.	Moderate	n.r.	11 × 9 mm hyperechogenic area near LCA origin; RV mid-cavity gradient 28 mmHg
6, after ECMO	≈42	45/20.7	18.2/14.5	10.5/5.5	Mild to moderate	37 (Vmax 3.0 m/s)	Paradoxical septal motion; TAPSE 3.6 mm; E/e′ 12.5 (septal), 19.3 (lateral)
9, ventilated	≈50	67.3/34.9	19.5/12.7	8.4/7.0	Mild to moderate	n.r.	TAPSE 5.2 mm; E/e′ 20.2 (septal), 24.9 (lateral)
13, ventilated	≈45	53.5/26.1	25.2/18.6	8.6/5.2	Moderate	22–25	TAPSE 5.6 mm
16, reintubated	37–40	34.0/15.2	25.2/21.4	6.6/5.8	Moderate	22–25	Levosimendan on day 15; TAPSE 4.1 mm
20	n.r.	48.2/22.9	23.8/18.4	7.5/6.6	Trace	26	Flat or paradoxical septal motion
21	n.r.	n.r./34	16/n.r.	n.r./up to 12	n.r.	n.r.	Marked LVPW thickening; E/e′ 20; TAPSE 5 mm; pulmonary acceleration time 50–70 ms
27	45–50	77 ^b^/n.r.	19/10	n.r./5 (9–11 ^c^)	Mild	20	Hyperechogenic endocardium and papillary muscles; E/e′ 7 (septal), 8 (lateral); E/A reversal
40, before discharge	n.r.	n.r.	21/n.r.	n.r./thickened	Trace	n.r.	Hyperechogenic endocardium and papillary muscles; E/e′ 8 (septal), 17 (lateral); TAPSE 10 mm; good flow in LM and LAD

Day 0 is the day of cardiac arrest and admission; surgical repair and institution of VA-ECMO took place on day 1. Echocardiographic studies on days 8, 19, 24 and 29 are shown only in Figure 1. ^a^ Calculated from the reported dimensions (day 0 only); all other values as reported. ^b^ Based on approximate dimensions (19/10 mm). ^c^ Measured in the apical four-chamber view. ^d^ M-mode EF (Teichholz formula) is shown as reported, for completeness only; it is not interchangeable with the Simpson EF and overestimates function in the presence of regional wall-motion abnormalities, flat or paradoxical septal motion and a small, thick-walled cavity. MR was graded qualitatively on color Doppler in all reports; quantitative indices were not reported. On every report from day 2 to day 20, the reimplanted LCA was described from its aortic origin along its epicardial course to the apex with preserved color Doppler flow (mid-course diameter approximately 3 mm from day 6); flow in the LCA was also documented on day 27, and the hyperechogenic area near the LCA origin was not described after day 5. Left atrial-to-aortic root ratio: 1.58 (day 13), 1.47 (day 16) and 1.22 (day 19). E/A, ratio of early to late mitral inflow velocity; E/e′, ratio of early mitral inflow velocity to early diastolic mitral annular velocity; ECMO, extracorporeal membrane oxygenation; EF, ejection fraction; FS, fractional shortening; IVSd, interventricular septum in diastole; LAD, left anterior descending artery; LCA, left coronary artery; LM, left main coronary artery; LV, left ventricle; LVEDd, LVESd, left ventricular end-diastolic and end-systolic dimensions; LVPW, LVPWd, left ventricular posterior wall (in diastole); MPA PG, main pulmonary artery peak gradient; MR, mitral regurgitation; n.r., not reported; RCA, right coronary artery; RV, right ventricle; TAPSE, tricuspid annular plane systolic excursion; VA-ECMO, venoarterial extracorporeal membrane oxygenation; Vmax, peak velocity.

**Table 3:** Differential diagnosis of severe left ventricular dysfunction in early infancy.

Feature	ALCAPA	Idiopathic Dilated Cardiomyopathy	Acute Myocarditis	Primary Endocardial Fibroelastosis
Typical presentation	Heart failure, feeding-related distress (infant angina), infarction or arrhythmia at 2–3 months after a silent neonatal period [2]	Heart failure at any age; familial, genetic or metabolic causes	Viral prodrome common; heart failure, arrhythmia or shock; fulminant course possible [29]	Heart failure in fetal life or infancy [30]
Electrocardiogram	Pathological Q waves in I, aVL, V5–V6 in about 66%; ST-T changes; normal in 8% [28]	LV hypertrophy or nonspecific ST-T changes; Q waves uncommon	Low QRS voltage; ST-T changes; conduction disturbances; arrhythmias [29]	LV hypertrophy and repolarization changes
Coronary arteries on echocardiography	No aortic origin of the LCA; reversed LCA flow into the PA (91%); collateral flow (85%); dilated RCA (81%) [6]	Normal origins and size	Normal	Normal in primary EFE; secondary EFE may accompany ALCAPA [30]
Myocardium and mitral valve	Regional wall-motion abnormalities; hyperechogenic papillary muscles and endocardium; ischemic MR [2]	Global hypokinesis; functional MR	Global or regional dysfunction; wall edema; pericardial effusion [29]	Diffusely thickened, hyperechogenic endocardium; globular LV [30]
Cardiac troponin	Markedly increased in acute infarction	Normal or mildly increased	Increased [29]	Normal or mildly increased
Confirmation	Echocardiography; CT angiography or catheterization if inconclusive [2,3]	Diagnosis of exclusion; genetic and metabolic testing	Cardiac MRI; endomyocardial biopsy [29]	Echocardiography; cardiac MRI; histology [30]
Present case	65 days of age; RCA 2.5 mm; no flow from the aorta into the LCA; hyperechogenic posterior papillary muscles (postoperative); hs-TnT 2620 ng/L	–	–	–

ALCAPA, anomalous left coronary artery from the pulmonary artery; CT, computed tomography; EFE, endocardial fibroelastosis; hs-TnT, high-sensitivity troponin T; LCA, left coronary artery; LV, left ventricle; MR, mitral regurgitation; MRI, magnetic resonance imaging; PA, pulmonary artery; RCA, right coronary artery. A dash indicates not applicable.

## Data Availability

The original contributions presented in this study are included in the article. Further inquiries can be directed to the corresponding author, subject to the consent of the patient’s parents.

## Abbreviations

The following abbreviations are used in this manuscript:

AHA	American Heart Association
AKI	acute kidney injury
ALCAPA	anomalous left coronary artery from the pulmonary artery
ALT	alanine aminotransferase
Ao	aorta
APTT	activated partial thromboplastin time
AST	aspartate aminotransferase
CK	creatine kinase
CK-BB	creatine kinase BB isoenzyme
CK-MB	creatine kinase MB isoenzyme
CPR	cardiopulmonary resuscitation
CRRT	continuous renal replacement therapy
CT	computed tomography
DIC	disseminated intravascular coagulation
E/A	ratio of early to late mitral inflow velocity
ECMO	extracorporeal membrane oxygenation
E/e′	ratio of early mitral inflow velocity to early diastolic mitral annular velocity
EF	ejection fraction
EFE	endocardial fibroelastosis
ELSO	Extracorporeal Life Support Organization
EMS	emergency medical service
FiO_2_	fraction of inspired oxygen
FS	fractional shortening
hs-TnT	high-sensitivity troponin T
ISTH	International Society on Thrombosis and Haemostasis
IVSd	interventricular septum in diastole
LAD	left anterior descending artery
LCA	left coronary artery
LGE	late gadolinium enhancement
LM	left main coronary artery
LV	left ventricle
LVEDd	left ventricular end-diastolic dimension
LVEF	left ventricular ejection fraction
LVESd	left ventricular end-systolic dimension
LVPW	left ventricular posterior wall
LVPWd	left ventricular posterior wall in diastole
MCS	mechanical circulatory support
MCV	mean corpuscular volume
MPA	main pulmonary artery
MPA PG	main pulmonary artery peak gradient
MR	mitral regurgitation
MRI	magnetic resonance imaging
n.r.	not reported
NT-proBNP	N-terminal pro-B-type natriuretic peptide
OHCA	out-of-hospital cardiac arrest
PA	pulmonary artery
PT	pulmonary trunk
PVR	pulmonary vascular resistance
RA	right atrium
RCA	right coronary artery
ROSC	return of spontaneous circulation
RV	right ventricle
TAPSE	tricuspid annular plane systolic excursion
URL	upper reference limit
VA-ECMO	venoarterial extracorporeal membrane oxygenation
Vmax	peak velocity
